# Current Concepts of ARDS: A Narrative Review

**DOI:** 10.3390/ijms18010064

**Published:** 2016-12-29

**Authors:** Michele Umbrello, Paolo Formenti, Luca Bolgiaghi, Davide Chiumello

**Affiliations:** 1 Unità Operativa Complessa di Anestesia e Rianimazione, Ospedale San Paolo—Azienda Socio Sanitaria Territoriale Santi Paolo e Carlo, 20124 Milano, Italy; michele.umbrello@fastwebnet.it (M.U.); formenti.paolo80@gmail.com (P.F.); 2 Dipartimento di Scienze della Salute, Università degli Studi di Milano, 20124 Milano, Italy; luca.bolgiaghi@hotmail.it

**Keywords:** acute respiratory distress syndrome, positive end-expiratory pressure, lung-protective ventilation, critically ill patients

## Abstract

Acute respiratory distress syndrome (ARDS) is characterized by the acute onset of pulmonary edema of non-cardiogenic origin, along with bilateral pulmonary infiltrates and reduction in respiratory system compliance. The hallmark of the syndrome is refractory hypoxemia. Despite its first description dates back in the late 1970s, a new definition has recently been proposed. However, the definition remains based on clinical characteristic. In the present review, the diagnostic workup and the pathophysiology of the syndrome will be presented. Therapeutic approaches to ARDS, including lung protective ventilation, prone positioning, neuromuscular blockade, inhaled vasodilators, corticosteroids and recruitment manoeuvres will be reviewed. We will underline how a holistic framework of respiratory and hemodynamic support should be provided to patients with ARDS, aiming to ensure adequate gas exchange by promoting lung recruitment while minimizing the risk of ventilator-induced lung injury. To do so, lung recruitability should be considered, as well as the avoidance of lung overstress by monitoring transpulmonary pressure or airway driving pressure. In the most severe cases, neuromuscular blockade, prone positioning, and extra-corporeal life support (alone or in combination) should be taken into account.

## 1. Introduction

Acute respiratory distress syndrome (ARDS) is defined by association of an acute onset of hypoxaemia and bilateral pulmonary infiltrates following a trigger insult; since its first description, ARDS has been redefined several times to ameliorate the accuracy of clinical diagnosis [1,2,3]. The first known description of the syndrome dates back to 1821, when Laennec described fatal “idiopathic pulmonary edema”. Later on, the first and the second World wars provided evidence that several traumatic insult could result in the eventual development of an edematous lung injury [4], so that and the term “shock lung” was developed to describe such a condition. In 1967, Ashbaugh and colleagues published a case-series of 12 patients that developed respiratory failure after a variety of insults [1], providing the first systematic description of this condition.

Nowadays, approximately 5% of hospitalized, mechanically ventilated patients meet the diagnostic criteria for ARDS [5]. As for the severity of the clinical presentation, it has been shown how only 25% of patients have a mild form of ARDS, while the remaining 75% display a moderate or severe form [6]. Indeed, in the last decades the incidence of ARDS has constantly declined, the change being primarily due to a reduction in the nosocomial form of ARDS [7], while the incidence of the community-acquired form has not changed. While this trend can partially be explained by a decrease in the routine use of chest radiographs [8] and arterial blood gas analyses [9], thus potentially leading to some degree of underdiagnosis, several major advances in critical care practice likely also contributed to this trend. Among key contributing measures are timely resuscitation and antimicrobial administration, restrictive transfusion strategies [10], ventilator care bundles [11], and the widespread use of lung-protective ventilation [12].

## 2. Definition

In 1994, during the American-European Consensus Conference (AECC) on ARDS, the term ARDS firstly achieved a common definition. Indeed, due to some critical issues of that definition, the European Society of Intensive Care Medicine convened an international expert panel in 2011 in Berlin, to develop a new definition of the syndrome, which led to the so-called “Berlin definition” of ARDS [3] (Table 1). According to this new definition, ARDS is an acute form of diffuse lung injury occurring in patients with a predisposing risk factor, meeting the following criteria: (1) onset within 1 week of a known clinical insult or new/worsening respiratory symptoms; (2) presence of bilateral opacities on chest X-ray, not fully explained by effusion, lobar/lung collapse, or nodules; (3) diagnosis of respiratory failure not fully explained by cardiac failure or fluid overload; (4) presence of hypoxemia, as defined by a specific threshold of the PaO_2_/FiO_2_ ratio measured with a minimum requirement of PEEP ≥ 5 cm H_2_O, thus identifying three categories of severity: mild (200 millimeters of mercury (mm) Hg < PaO_2_/FiO_2_ ≤ 300 mm Hg), moderate (100 mm Hg < PaO_2_/FiO_2_ ≤ 200 mm Hg), severe (PaO_2_/FiO_2_ ≤ 100 mm Hg) [1,3].

Indeed, the Berlin criteria provided a little but significant improvement in the predictive ability for mortality (area under the curve AUC 0.577), when compared to the AECC criteria (AUC 0.536). However, some issues still remain with this definition, the bigger being the lack of a sensitive and specific biomarker that can help the clinical diagnosis. In fact, even if the several biomarkers are currently under investigation, they have not proven helpful enough to be introduced into clinical practice [13,14]. Moreover, the level of positive end-expiratory pressure applied may also greatly affect PaO_2_/FIO_2_ value, thereby masking acute respiratory distress syndrome severity, which should reflect the underlying lung injury (lung edema and recruitability). Recently, it has been demonstrated how the assessment of acute respiratory distress syndrome severity at standardized low positive end-expiratory pressure (i.e., 5 cm H_2_O) allows a better evaluation of lung recruitability and edema than at higher positive end-expiratory pressure clinically set [15]. Eventually, the role of excessive hydrostatic pressure, not always easily identified by the use of echocardiography or computed tomography (CT) scan, remains a major confounder.

## 3. Diagnostic Evaluation

Common risk factors for ARDS are: pneumonia, sepsis, gastric content aspiration, trauma, pancreatitis, inhalation injury, burns, non-cardiogenic shock, drug overdose, acute lung injury following massive tranfusions (TRALI), drowning [16]. Indeed, the critical factor for a favorable outcome of ARDS patients is an adequate treatment of the underlying cause. Undoubtedly, pneumonia still remains leading cause of ARDS [17], hence the first step is to quickly identify the pathogen responsible for the infection, and microbiological assessment for any potential pathogens represents the first diagnostic effort. Table 2 shows the microorganisms most often associated with a diagnosis of ARDS.

Community-acquired bacterial pneumonia represents the first type of pneumonia leading to ARDS [17]. Nosocomial bacteria, on the other side, should be considered in mechanically-ventilated or hospitalized patients who develop ARDS [18]. Indeed, a recent study found a 36% rate of viruses isolated from the respiratory tract of patients with ARDS, compared to a historic value as low as 5%–10% [19,20,21], the majority being respiratory viruses [22]. As viruses can cause pneumonia and ARDS, the suggested diagnostic technique is performing real-time PCR on a bronchoalveolar lavage (BAL) sample [23]. HSV and CMV are increasingly recognized causes of ARDS [24,25]. Moreover, fungi and parasites such as *Pneumocystis jirovecii*, *Toxoplasma gondii* and *Aspergillus fumigatus* may be responsible, in immunocompromised patient, for some forms of ARDS [26]. Prompt recognition is then pivotal, because a specific treatment may change the outcome [26].

**Figure 1:**
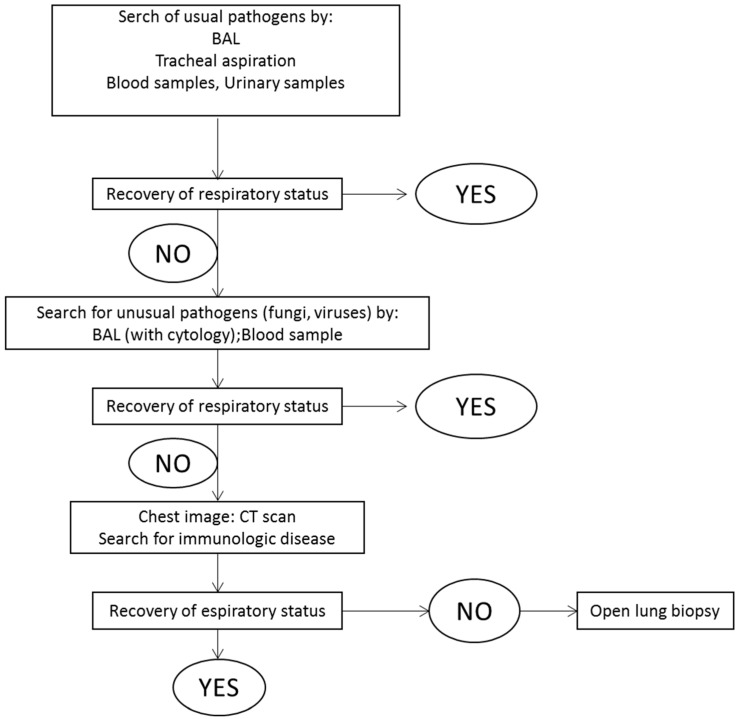
Diagnostic approach to identify the causal pathogen in patients with pulmonary ARDS.

Physicians caring for patients with ARDS thus need to first systematically investigate a potential infectious etiology, and such initial assessment should include: blood cultures, urinary antigen testing for *Legionella pneumophila* and *Streptococcus pneumoniae*, serologic tests for *Mycoplasma pneumoniae* and *Chlamydia pneumoniae*, and microbial sampling of the lung [27], preferably realized with a fiberoptic BAL. The first step aims at bacterial identification using Gram staining, while the following step aims at recognition of respiratory viruses using PCR. Indeed, a recent study investigated the prevalence of ARDS without any identifiable risk factor, demonstrating a prevalence of 7.5% of cases [28]. In this scenario, BAL cytology, CT scan and immunologic examinations should be performed, looking for less common etiologies. Drug-induced respiratory failure or a malignant etiology should also be considered. Eventually, if neither CT scan nor BAL cytology help the clinicians in the diagnosis, open lung biopsy (OLB) should be performed to identify the underlying trigger cause. Another potential role for OLB is the histological identification of fibroproliferation, which occurs after the first week of evolution in a subset of patients, in order to consider the use of corticosteroids [29]. As previously discussed, then, there are in the current clinical practice only few indications to perform an OLB in patient with non-resolving ARDS [30]. Figure 1 shows a schematic diagnostic approach to identify the causal pathogen in patients with ARDS.

**Figure 2:**
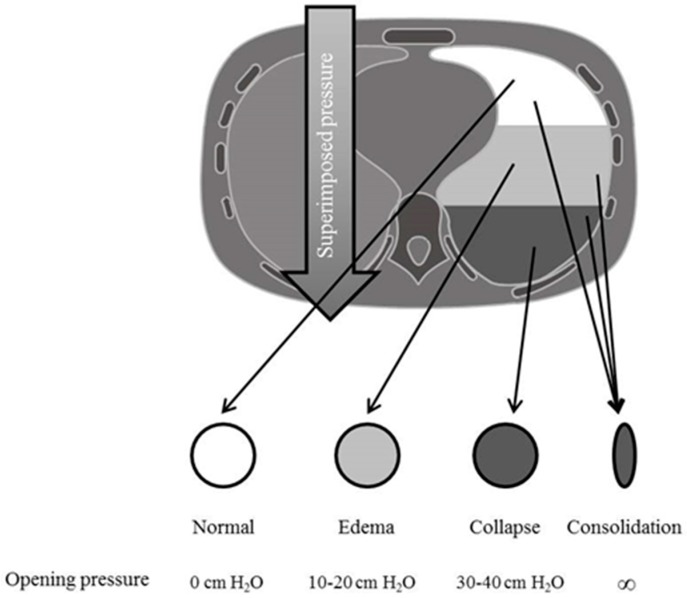
Ideal model depicting the effects of increased permeability in terms of increased superimposed pressure, with the inhomogeneous coexistence of areas of hyperinflation, normal inflation, collapse and areas of consolidation (as indicated by arrows), along with the necessary pressure that needs to be applied to the lung in order to overcome the superimposed pressure generated by the lung mass and by the chest wall and recruit the alveolar units (i.e., to inflate the collapsed lung regions) and to maintain these regions open. ∞ represents infinite pressure, i.e., areas that can never be open despite increased positive airway pressure.

**Figure 3:**
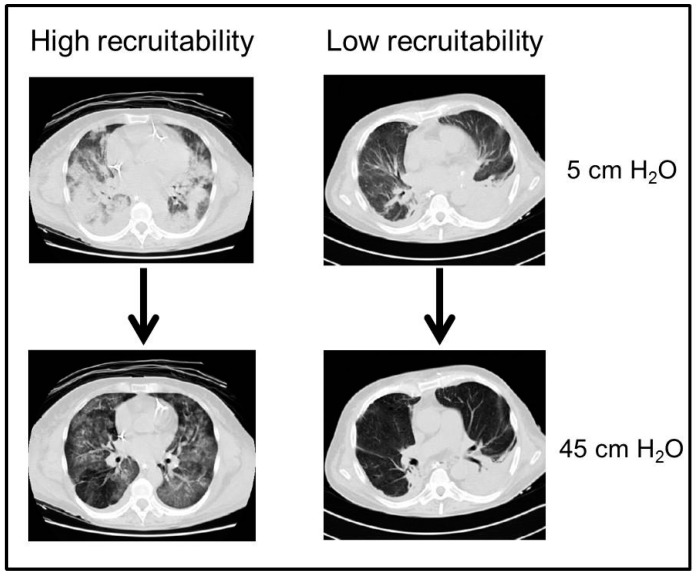
Example of lung CT scan of patients with high (**upper** panel) or low (**lower** panel) potential of lung recruitment. Arrows depict the morphologic change from a condition of low airway pressure (i.e., 5 cm H_2_O), to one of high airway pressure (i.e., 45 cm H_2_O).

A pulmonary CT scan is also usually performed to better understand the underlying patophysiology and the possible presence of hidden diagnosis; typical morphological patterns are: consolidated regions (homogeneous areas of increased density without vessels and bronchi), ground glass areas (with augmented density but still recognizable vessels) and normally aerated regions [31]. Consolidated regions are typically localized on the dependent areas of the lung; they are related to the increase in lung weight, due to the presence of lung oedema, so that the increased superimposed pressure determines a reduction of lung gas volume and the development of non-aerated regions [32] (Figure 2). Moreover, CT scan has been used to evaluate the extent of lung recruitability, defined as the aeration of previously collapsed or non-aerated lung units following an increase in alveolar pressure. With this technique, lung recruitability was found to be highly variable among patients with ARDS, with values ranging from 0 to 70% of total lung weight, as shown in Figure 3. The presence of non-inflated areas determines a major expansion of the neighboring lung regions, causing an increase in the local pressure and thus acting as a “stress raiser” [33]. Typically, pulmonary ARDS present a similar amount of consolidated and ground glass areas, while extrapulmonary ARDS have a higher amount of ground glass areas [34]. CT scan may also be helpful in describing the distribution of lung opacities and, in some instances, it may allow the recognition of an unsuspected pneumothorax or help to identify the ARDS cause.

**Figure 4:**
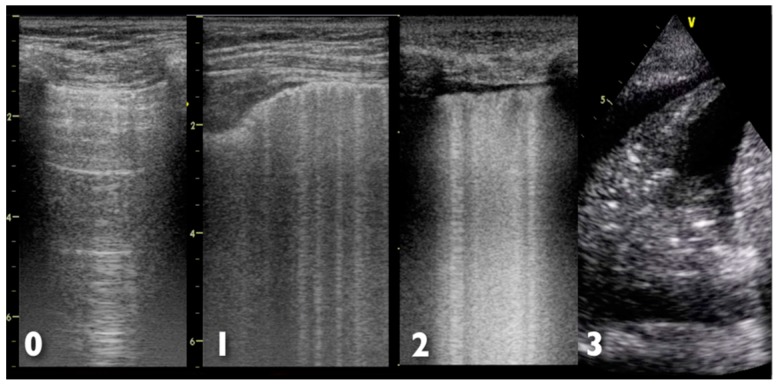
Possible ultrasonographic findings at lung examination. **0**: Normal aeration with normal sliding, with A-lines pattern; **1**: Multiple B-lines but separated by at least 5 mm; **2**: Multiple, coalescent, not well-separated B-lines; **3**: Lung consolidation, hyperechoic area with air bronchogram. Numbers on the left side of each ultrasound image represent the depth (in cm).

Further help in diagnosis and management of ARDS may come from the study of ultrasonographic artefacts produced by air, lung parenchyma, chest wall and pleura [35,36]; the pivotal finding in patients with respiratory failure is a B-line artifact, defined as the presence of a discrete vertical hyperechoic reverberation artefact that arises from the pleural line [36]. The finding of three or more B-lines in one intercostal space is considered abnormal and is referred to as a B-pattern [36]. The interstitial involvement of the lungs correlates with the presence of B-lines, and a preponderance of B-pattern is suggestive of an alveolar process, rather than a non-alveolar cause [37]. However, the presence of a bilateral B-pattern does not permit a differentiation between ARDS and cardiogenic pulmonary oedema [38]. Indeed, in ARDS, more commonly than in cardiogenic pulmonary oedema, a non-homogeneous distribution of B-pattern, C (consolidative) pattern and pleural line abnormalities are observed [39]. The systematic use of thoracic ultrasonography as a tool for bedside evaluation of ARDS evolution has been suggested by some authors [40,41]. Figure 4 shows the different ultrasonographic finding of lung examination; ultrasonographic findings in cardiogenic pulmonary oedema and ARDS are summarized in Table 3.

## 4. Patophysiology

The main characteristic of ARDS is an increased pulmonary capillary permeability. The consequent accumulation of protein-rich fluid inside the alveoli is the result of the damage to the capillary endothelium and alveolar epithelium; this cause the release of cytokines, producing diffuse alveolar damage [42]. Since the lung is composed by two type of alveolar epithelial cell, damage to type I cells leads to an increase of fluid entry into the alveoli and a decrease of fluid clearance; on the other side, damage to type II cells results in a diminished production of surfactant that cause a compliance reduction and alveolar collapse. In the lungs of patients with ARDS several abnormalities have been found that involve gene transcription for pro-inflammatory mediators [43]; moreover, a relationship between the systemic response to endotoxin and the induction of cyclo-oxigenase-2 gene expression has been suggested [44].

The characteristic pathological features of ARDS have classically been described by three overlapping phases: an exudative or inflammatory phase, a proliferative phase and a fibrotic phase. However, other variables, such as the occurrence of nosocomial pneumonia or ventilator induced lung injury (VILI), may complicate these sequence. The initial fluid accumulation is followed, within 72 h, by a variable amount of proliferation of type II alveolar cells, fibroblasts and new matrix deposition. Patient who develop fibrosis show a reduction in pulmonary compliance, further worsening in gas exchange and increased mortality [45]; indeed, the reason why some patient progress to fibrosis whereas other progress toward resolution is not completely understood [46]. Similarly, the pathophysiologic link between ARDS and the following development of multiple organ failure, which often is the ultimate cause of death, is not completely understood [47].

Based on our current knowledge, a lung protective ventilatory strategy has been developed, which insures adequate oxygenation and CO_2_ clearance, furthermore minimizing the extent of the damage due to the institution of mechanical ventilation (VILI) [48]. Over time, VILI has been attributed to excessive stress, tidal volume [49], driving pressure [50], respiratory rate and gas flow [51]. Recently, a unifying theory has hypothesized that the fundamental determinant of VILI may be the result of an excessive mechanical power applied to the lungs [52,53,54]. As said, an inappropriate ventilatory strategy can exacerbate the initial lung injury, both in terms of excessive stress consequent on the excessive volume or pressure with which the lungs are ventilated, but also as inappropriately low levels of PEEP may cause the repetitive opening and closing of alveoli, which in turn exacerbates the proinflammatory response [55]. The still actual framework is that of the “baby lung”, first introduced by Gattinoni [56], which models the lung of a patient with ARDS as a small aerated lung; starting from the consideration that respiratory system compliance is linearly related to the “baby lung” dimensions, the author suggested that the ARDS lung is not “stiff” but instead small, with nearly normal intrinsic elasticity. Moreover, the density redistribution in prone position shows that the “baby lung” is a functional and not an anatomical concept. The size of the baby lung determines the lung susceptibility to VILI, so that the smaller the baby lung, the greater is the potential for unsafe mechanical ventilation.

## 5. Treatments

The primary targets for ARDS treatment are to ensure adequate gas exchange while minimizing the risk of VILI. Indeed, to date, the treatment remains largely supportive. Different, both pharmacologic and non-pharmacologic, strategies exist to reach this objective and several types of mechanical ventilatory support may be provided. Table 4 provides a summary of the treatment strategies described.

### 5.1. Non-Pharmacologic Interventions

#### 5.1.1. Non-Invasive Ventilation

Non-invasive ventilation (NIV) could reduce the work of breathing and the extent of intrapulmonary shunt, thereby improving gas exchange, with the advantage of avoiding deep sedation and lowering the risk of nosocomial pneumonia; however, its use is still under debate because of the high risk of failure and the possible consequent risk of delaying tracheal intubation and invasive mechanical ventilation. Recently, a meta-analysis, based on 13 studies with 540 patients treated with NIV, showed an intubation rate varying between 30% and 86% and a mortality rate ranging from 15% to 71% [57]. However, as the majority of these studies were not randomized, is not possible to extrapolate firm conclusions. Given the high risk of failure, NIV should be provided in a strictly monitored environment such an intensive care unit and should be reserved to patient without extra-lung involvement.

The recent introduction of high flow nasal cannulae (HFNCs) could represent a valid alternative to NIV. This device can deliver a high oxygen flow through the nose, yet delivering sufficient heating and humidity [58]; it proved able to reduce the work of breathing, to improve oxygenation and CO_2_ clearance, and to increase the end expiratory lung volume. A recent observational study in ARDS patients [59], show a 40% failure rate, with subsequent endotracheal intubated; however, this finding was similar to the 46% found by by Antonelli et al. in a study of NIV in ARDS [60]. Currently, only one randomized study compared HFNCs, NIV and oxygen therapy in acute respiratory failure [61]; the results show hoe there is no difference between the three groups with respect to the intubation rate, while in the high flow nasal cannula group intensive care unit mortality was lower.

#### 5.1.2. Invasive Mechanical Ventilation

Mechanical ventilation represents a supportive therapy able to guarantee sufficient gas exchange, providing both an increase in PaO_2_ and CO_2_ removal, while reducing respiratory muscle activity [62]. The effect of mechanical ventilation on oxygenation is twofold: first, it allows the titration of FiO_2_; secondly, it provides, during the inspiratory phase, enough positive pressure to ensure the opening of collapsed pulmonary units. However, without the application of an appropriate level of positive end-expiratory pressure (PEEP), the same pulmonary units will collapse again during the expiratory phase [63].

Indeed, a completely “safe” ventilatory strategy does not exist, and the support must be tailored to each single patient, based on hemodynamics, gas exchange, lung recruitability and respiratory mechanics. The last 30 years of literature show how the use of high-volume and high-pressure ventilation can damage the lung [64]: ventilatory strategies characterized by high-volume may cause both the development of pulmonary edema in the uninjured lung [49,65] and the worsening of that in the injured lung [66,67]. These effects are primarily due to alveolar overdistention, which in turn causes endothelial and epithelial injury, then promoting a proinflammatory cascade. The same proinflammatory cascade is also promoted by the continuous alveolar collapse and reopening, the so-called atelectrauma [68].

**Figure 5:**
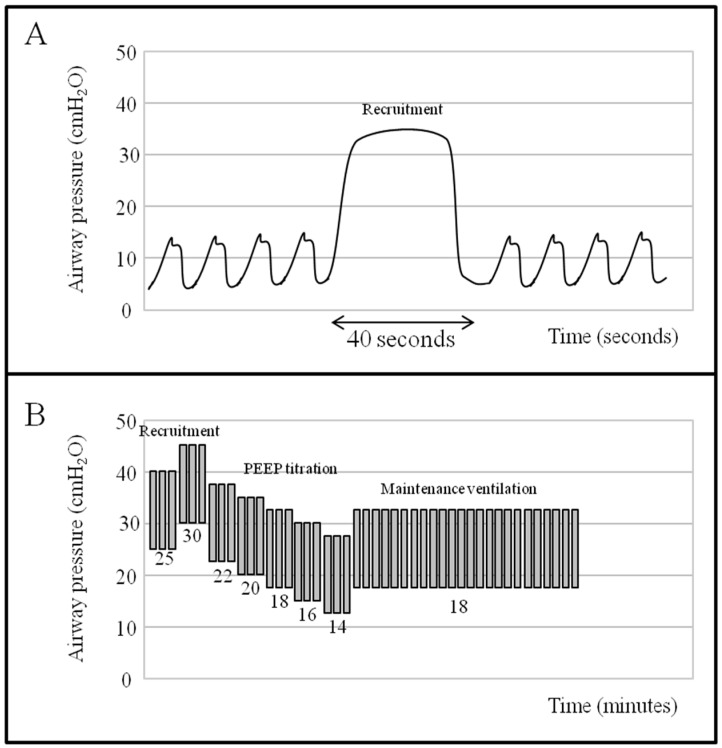
Pressure-time curve showing different recruitment maneuvers. (**A**) sustained inflation sigh using continuous positive airway pressure (CPAP) of 35 cm H_2_O for 40 s (as depicted by the arrow); (**B**) stepwise recruitment maneuver using both plateau pressure and PEEP increase, keeping a fixed driving pressure of 15 cm H_2_O; after recruitment, a decremental PEEP titration is performed until an optimal level is identified (e.g., one associated with the best compliance or best oxygenation).

Since a decrease in alveolar inhomogeneity was shown to reduce the VILI [69], the application of high PEEP levels, while opening the collapsed alveoli and decreasing the intrapulmonary shunt, might decrease the repetitive alveolar opening and closing during the whole respiratory cycle [70]. However, when two different large RCTs were performed to compare ARDS patients treated with low vs. high levels of PEEP [71,72], the results did not demonstrate any benefit of a high PEEP strategy. This apparently contradictory finding may be interpreted by considering the concept of lung recruitment, defined as the extent of the collapsed regions in which aeration can be restored with increasing airway pressure. In order to recruit and maintain a lung region open, the pressure generated by the lung mass and by the chest wall, named superimposed pressure, must be overcome [73]. Various techniques exist to recruit the lung, such as the sigh (a high tidal volume intermittently delivered during ventilation), the extended sigh (a stepwise increase of PEEP or both PEEP and plateau pressure) and the sustained inflation (a static increase in airway pressure applied for 20–40 s) [74] (Figure 5). The main target, irrespective of the technique used, is to apply a high transpulmonary pressure for an adequate time, so to cause the reinflation of the closed pulmonary units. While these maneuvers are able, without major side effects, to improve oxygenation for a variable period of time, however their use has not shown per se to lead to a significant reduction in mortality [75].

#### 5.1.3. Lung Recruitment

Lung recruitment is defined as the enrollment of pulmonary units in a new status of inflation [76]. In patients with ARDS, a varying extent of lung recruitability was found, ranging from 0% to 70% of the total lung weight as estimated by lung CT-scan [77]. Pulmonary CT-scan is the gold standard for the measurement of lung recruitability, although it requires the transport of the patient outside the ICU and the use of X-rays [78]. As an alternative, lung ultrasound proved reliable in estimating lung recruitability at the bedside, but further studies are necessary to confirm this finding [79].

In order to re-inflate the collapsed lung regions, it is necessary to overcome the superimposed pressure generated by the lung mass and by chest wall. A transient increase in inspiratory airway pressure to 40–45 cm H_2_O is generally used for this aim. Different types of recruitment maneuver, such as sustained inflation, intermittent sighs and stepwise increase in inspiratory pressure, have been suggested [74]. Indeed, the optimal procedure has not yet been defined. Independently of the specific maneuver applied, oxygenation improves for a certain period of time without major side effects; however, recruitment maneuvers alone were not associated to a reduction in the mortality [75].

#### 5.1.4. PEEP Selection

The selection of the ideal level of PEEP is an issue hard to resolve: if PEEP is too low some portion of recruitable tissue will collapse, whereas excessive PEEP generate dead space and tissue stretch. The philosophy behind the application of PEEP has changed over time: while in the sixties it was considered as a tool to improve oxygenation, it is now regarded as a key element to avoid the repetitive alveolar opening and closing during the respiratory cycle, so that it reached a prominent position in the framework of lung protective ventilation [16,70,80,81]. Indeed, the key question is how to titrate PEEP on individual patients. Various approaches have been proposed to set PEEP (Table 5); the most commonly used is titration based on a PEEP/FiO_2_ table using as a target the level of saturation/oxygenation [72]. However, it should always be kept in mind that the improvement in oxygenation can simply be due to a hemodynamic effect (i.e., the reduction of cardiac output and right-to-left shunt) without any effect on lung recruitment. Another method is based on respiratory mechanics, with the aim of keeping airway pressure under a safe limit (26–28 cm H_2_O), through stepwise increase of PEEP while maintaining a constant tidal volume [82]. Talmor et al. [83] showed improved compliance and oxygenation when PEEP was set according to an absolute level of end-expiratory transpulmonary pressure between 0 and 10 cm H_2_O. Other authors used the tidal variation in esophageal pressure, rather than its absolute value, to evaluate the total end-inspiratory transpulmonary pressure, then used as a marker of lung stress [84]. Given the difficulty to choose the optimum PEEP level, which can simultaneously guarantee the higher level of oxygenation, the higher compliance and the lower overdistention, then possibly reducing the risk of VILI, we recommend to stratify ARDS severity by ventilating the patient at PEEP 5 cm H_2_O in pure oxygen, as suggested [15]. In case of severe (or moderate-to-severe) ARDS, lung recruitability should be computed by lung CT scan or ultrasound, and high PEEP levels (i.e., >15 cm H_2_O) should be applied. In addition, to avoid lung overstress, transpulmonary pressure should be measured while simultaneously titrating PEEP and tidal volume.

#### 5.1.5. Tidal Volume Setting

The main determinant of VILI is the ratio between the size of tidal volume and that of the resting lung volume in which it is distributed: together, they determine the non-physiologic stress (tension generated within the lung tissue) and strain (deformation of the lung) [88]. Then, to maintain a low stress and strain we need a low tidal volume or a high resting volume [82,83]. A seminal study on ventilator strategy in ARDS (the ARMA trial), demonstrated how using a tidal volume of 6 mL/kg (predicted body weight), as compared to the then conventional setting of 12 mL/kg, a 22% reduction in mortality could be achieved [89]. A recent meta-analysis confirmed those findings, showing a significant reduction in 28-day mortality in patient treated with the so-called “lung-protective ventilation” [90]. Despite these data, which have been available for as much as two decades, the use of low tidal volume ventilation is still not ubiquitous [91]. To conclude, in patients undergoing mechanical ventilation, the use of an excessive tidal volume increases the risk of developing ARDS, while the exposure to high tidal volumes in patients with established ARDS increases mortality.

Of note, since actual body weight is not an accurate index of lung size, the use of predicted body weight (based on height and sex) is currently recommended to calculate the appropriate tidal volume. However, even predicted body weight is poorly related to the resting volume, to the extent that a similar tidal volume can generate different lung stress/strain [92]. With the aim of better individualizing the tidal volume, the use of airway driving pressure has recently been proposed [50]. The latter, i.e., the ratio between tidal volume and respiratory system compliance, should in fact better reflect lung stress/strain, as the respiratory system compliance is related to the amount of lung gas volume [93]. Recently, Amato et al. found that in a pooled sample of >3500 ARDS patients ventilated with different combinations of tidal volume and PEEP, the airway driving pressure was the factor most associated with the outcome: a higher mortality was only found when higher plateau pressures were observed in patients with higher driving pressures. Similarly, the protective effects of higher PEEP was only seen when this was associated with a decreased driving pressures, with a cutoff for increased mortality at a driving pressure of 15 cm H_2_O [50]. Nonetheless, the driving pressure has limitations, the main being that transpulmonary pressure, and not airway pressure, is the relevant distending pressure for the lung. This is significant as the chest wall has been as unpredictably altered in ARDS [94]. Indeed, the measurement of functional residual capacity, as an index of the baby lung size, seems more physiologically appropriate, and its use may open the route to new studies that may further optimize tidal volume setting. Indeed, a recent paper showed how airway driving pressure can detect lung overstress with an acceptable accuracy patients with ARDS, as those with higher airway driving pressure group had a significantly higher lung stress, respiratory system and lung elastance as compared to those with lower airway driving pressure [95].

While in the past years the choice of a specific mode of mechanical ventilation (i.e., pressure-controlled versus volume-controlled), was considered relevant for patient outcome, two recent meta-analysis were not able to show any significant difference in mortality, risk of barotrauma or other physiologic responses (cardiac output, gas exchange, work of breathing) [96,97].

#### 5.1.6. Oxygen and Carbon Dioxide Target

As recently demonstrated by Panwar et al. [98], similar outcomes and number of organ failures were found in patients with ARDS randomized to an arterial oxygen saturation target >96% or between 88% and 92%. The actual recommendation is a conservative oxygenation strategy with an arterial oxygen saturation target between 88% and 95% in patients receiving invasive mechanical ventilation. Indeed, the use of a low tidal volume, with the aim to reduce the risk of VILI, may cause the development of hypercapnia. However, arterial carbon dioxide levels up to 70 mm Hg with a pH of 7.20 were found to be safe [99,100], in the absence of pathological condition such as raised intracranial pressure or right heart failure. The rationale of a more liberal CO_2_ management (permissive hypercapnia) lies in the well-known positive effects of hypercapnic acidosis on arterial and tissue oxygenation: the potentiation of hypoxic pulmonary vasoconstriction, the inhibition of airway tone, the increase in cardiac output, the anti-inflammatory effect and the rightward shift in the oxygen-hemoglobin dissociation curve [101].

#### 5.1.7. Prone Positioning

As in the case of PEEP, the use and indications of prone positioning in patients with ARDS has changed over time. While decades ago this procedure was only used to improve arterial oxygenation in life-threatening acute respiratory failure [102,103], it is nowadays clear that prone positioning, allowing for a more homogeneous distribution of stress and strain, helps to protect lung against the VILI [104]. The most important consequences of prone positioning, which can explain its final effect are: a better ventilation/perfusion matching with a consequent improvement in CO_2_ clearance, a more homogenous distribution of ventilation with a reduction of VILI and a recruitment of dorsal regions through the redistribution of lung densities [104,105]. Therefore, prone positioning should be reserved to all patients with severe ARDS, especially in the acute phase, because of the higher probability to recruit lung parenchyma [37].

Given these premises, a multicenter randomized trial was designed to evaluate the use of prone positioning in severe ADRS, for a minimum of 16 h per day (the Proning Severe ARDS Patients (PROSEVA) trial) [105]. The study showed a higher extubation success and a significant reduction in 28-day mortality in the prone positioning-group (16% vs. 32%). Indeed, the simultaneous use of prone positioning and NMBAs could exert a synergistic effect on oxygenation and decreasing the duration of mechanical ventilation, eventually improving the final outcome. The few absolute contraindications to prone positioning that have to be taken into consideration are: pregnancy, hemodynamic instability, open abdomen treatment and unstable fractures [104].

#### 5.1.8. Extracorporeal Assistance

The use of extracorporeal membrane oxygenation (ECMO) for the treatment of ARDS was introduced in the early 70‘s with the aim of guaranteeing a protective ventilation and minimizing the risk for the VILI, as an artificial lung may provide an adequate blood CO_2_ removal and oxygenation, allowing to reduce mechanical ventilation. Several observational studies demonstrated some degree of benefit from the use of ECMO (Venteruolo); recently, a randomized trial (CESAR study) of patients with ARDS referred to an ECMO center showed a higher 6-months survival rate (63% vs. 47%) and no difference in quality of life and spirometric parameters compared to patients treated with conventional mechanical ventilation [106]. In spite of these positive data, the CESAR trial has been criticized for its design; therefore, currently, is not possible to conclude for a superiority of ECMO with respect to conventional mechanical ventilation [107].

### 5.2. Pharmacologic Interventions

#### 5.2.1. Myoresolution

When a patient show a high ventilation demand, as in the event of ARDS, his vigorous breaths might generate a transpulmonary pressure too high to ensure lung protective ventilation; in this case, spontaneous breathing could worsen the extent of lung damage [48]. Moreover, during intense spontaneous breathing, the negativization of pleural pressure brings to an increase of venous return and hence of cardiac filling pressures that may increase the risk of VILI by itself [81].

Whit the purpose to ameliorate patient-ventilator synchrony and to reduce the oxygen consumption related to respiratory muscle activity, many clinicians decide to abolish any spontaneous respiratory effort by using neuromuscular blocking agents (NMBAs) [99]. An additional effect of NMBAs is the reduction of the negative increase in pleural pressure seen during spontaneous breathing, with the likely consequent reduction of stress and strain applied to the lung [99]. On the other hand, NMBA use may lead to the development of diaphragmatic dysfunction or ICU-acquired weakness. Indeed, it has been shown how patients with severe ARDS treated with an early, short-course of NMBAs presented lower mortality, reduced length of mechanical ventilation and less episodes of barotrauma [12].

The current knowledge seems to suggest that in patients with severe ARDS spontaneous breathing seems to be dangerous, whereas it appears to be beneficial in patients with a mild to moderate form. The use of NMBAs should then be reserved to the most severe patients, in order to insure patient-ventilator synchrony and prevent the generation of a dangerously high transpulmonary pressure, while the need of pharmacological paralysis should be evaluated daily.

#### 5.2.2. Inhaled Vasodilators

Despite the well-known vasodilatory effects exerted by nitric oxide on the pulmonary vasculature, leading to an improved ventilation/perfusion matching, its use in ARDS patients is highly controversial [108], as no clear mortality benefit could be demonstrated. Moreover, its use was associated with important cost-safety concerns and an increase in the incidence of renal failure [109].

#### 5.2.3. Corticosteroids

The central role of the inflammatory response in the pathogenesis of ARDS is the rationale behind the idea to use corticosteroids as a therapy in ARDS patient. Based on these concepts, several trials investigated corticosteroids use [110,111], however with heterogeneous results. Meduri [110] in its study conducted in the early phase of ARDS demonstrated a decrease in ICU mortality rate; however these findings could not be replicated in other studies [111,112].

A possible explanation of these conflicting results may lie in a varying pathophysiology of the inflammatory state present in the different studies. MicroRNA (miRNA) are short, non-coding RNAs that pair to specific messenger RNA (mRNA) targets and negatively regulate gene expression [113]. miRNA have shown to regulate genes involved in normal lung physiology and inflammatory lung states [114]. A recent paper studied miRNA present in blood leukocytes of patients with ARDS during the first week of care, with a particular focus on the effects of corticosteroid therapy on miRNA expression during the first week of care [115]. The authors identified 21 miRNA that are expressed at increased levels at the onset of ARDS, remain elevated at day 3 and increase further by day 7, suggesting that the underlying inflammatory processes that led to ARDS remained active at day 3 and the enhanced miRNA expression by day 7 may have a role in the resolution of inflammation. Steroid therapy had no effect on the elevated miRNA species observed on days 3 or 7. These data suggest the presence of steroid-responsive and steroid-independent inflammatory axes during the course of ARDS, and that miRNA and corticosteroids may have similar but relatively independent mechanisms that modulate inflammation. The increased expression of miRNA, independent of corticosteroid therapy, may suggest a role in steroid-independent mechanisms that contribute to the resolution of inflammation, thus potentially explaining the different response to corticosteroid therapy seen in different patient cohorts.

## 6. Conclusions

ARDS still remains a syndrome with an elevated overall incidence, and with an attributable mortality ranging from 40% to 60%. To allow for a better accuracy of the clinical diagnosis, its definition has been reviewed several times, the last in Berlin, 2011. In order to ensure a rapid etiologic therapy, a rapid identification of the underlying cause is mandatory, and the use of a systematic approach to diagnosis may help the clinicians. Lung CT scan represents an important tool both for the diagnosis of extra-pulmonary causes of ARDS and for the evaluation of lung recruitability and the consequent ventilator setting. Ultrasonography had earned an important role in the bedside evaluation of lung parenchyma, in association with the assessment of left and right ventricular function. The supportive treatment of patients with ARDS should be oriented to sustain the vital functions, to improve and ensure an adequate gas exchange, while reducing the probability to cause damage such as by VILI. Irrespective of the mode of mechanical ventilation, lung recruitability should be assessed before setting the PEEP value, and an inspiratory O_2_ fraction should be chosen to target an arterial saturation between 88% and 95%. Lung volume and transpulmonary pressure monitoring might help to adjust the ventilator settings and to avoid lung overstress, while maintaining a lung-protective strategy. Eventually, the use of prone positioning and myoresolution should always be considered, at least in the most severe cases.

## Figures and Tables

**Table 1:** Comparison between AECC definition (1994), and the Berlin definition of acute respiratory distress syndrome (2012).

Characteristic	AECC Definition 1994 [2]	Berlin Definition 2012 [3]
Timing	Acute, without any specification	Maximum within a week after a trigger insult
Imaging	Chest X-ray with bilateral infiltrates	Chest X-ray or CT scan with bilateral infiltrates, not fully explained by effusion, lung collapse or nodules
Non-cardiogenic source of edema	Confirmation of non-elevated left atrial pressure	Respiratory failure not completely explained by excessive volume loading or cardiac failure
Classification	Based on PaO_2_/FiO_2_	Based on PaO_2_/FiO_2_ calculated with PEEP ≥5 cmH_2_O
	Acute lung injury: ≤300	Mild: 201–300
	ARDS: ≤200	Moderate: 101–200
	–	Severe: ≤100
Predisposing condition	Not specified	If none identified, then need to rule out cardiogenic edema with additional data

**Table 2:** Most common pathogens responsible for ARDS genesis.

Bacteria	Virus	Fungi	Parasites
*Streptococcus pneumoniae*	Influenza A and B	*Pneumocystis Jirovecii*	*Toxoplasma gondii*
*Haemophilus influenzae*	Rhinoviruses		
*Enterobacteriaceae*	RSV		
*Staphylococcus aureus*	Parainfluenza viruses		
*Legionella pneumophila*	Coronavirus		
*Clamydia pneumoniae*	Enterovirus	*Aspergillus fumigatus*	
*Mycoplasma pneumoniae*	HSV		
*Pseudomonas aeruginosa*	CMV		
*Acinetobacter baumannii*	–		
*Stenotrophompnas maltophilia*	–		

**Table 3:** Comparison between ultrasonographic findings in ARDS and cardiogenic pulmonary edema.

Condition	Thoracic Ultrasound	Cardiac Ultrasound
ARDS	Bilateral B pattern	No change in ventricular function vs. previous examination
	Non-uniform distribution	
	Pleural line abnormalities	No inferior vena cava dilation (diameter < 23 mm)
	Reduced in lung sliding	E/e’ ≤ 8
	C pattern	–
Cardiogenic Pulmonary Edema	Bilateral B pattern	New or worsening left ventricular disfunction
	Uniform distribution	Inferior vena cava dilation (≥23 mm)
	Pleural effusion	E/e’ ≥ 14
	Left-sided predominance	–

E/e’ represents the ratio between the peak early diastolic mitral velocity between the tips of mitral leaflets (E wave) and the spectral tissue Doppler-derived peak early diastolic velocity at mitral annulus (E’ wave), thus yielding an accurate estimate of lesft ventricular diastolic function.

**Table 4:** Pharmacologic and non-pharmacologic strategies for patients with ARDS.

Non-Pharmacologic	Pharmacologic
Non-invasive ventilation	Myoresolution
Invasive mechanical ventilation	
Lung recruitment	Inhaled vasodilators
PEEP selection	
Tidal volume setting	Corticosteroids
Oxygen and Carbon Dioxide target	
Prone positioning	–
Extracorporeal assistance	

**Table 5:** Methods for bedside PEEP selection.

Method	Characteristics
Lung Open Ventilation (LOV) study [72]	Setting PEEP as for the PEEP/FiO_2_ table of the lung open ventilation arm of LOV trial
ExPress [85]	Maintain an inspiratory plateau pressure between 28 and 30 cm H_2_O according to the increased recruitment strategy of the ExPress trial
Stress Index [86]	Obtain a stress index coefficient of 1
Esophageal pressure [87]	Setting PEEP targeting an absolute end-expiratory transpulmonary pressure of 0–10 cm H_2_O
